# The courage to change: Patient perceptions of 12-Step fellowships

**DOI:** 10.1186/1472-6963-11-339

**Published:** 2011-12-15

**Authors:** John-Kåre Vederhus, Christine Timko, Øistein Kristensen, Thomas Clausen

**Affiliations:** 1 Addiction Unit, Sørlandet Hospital HF, Kristiansand, Norway; 2 Center for Health Care Evaluation, Department of Veterans Affairs Health Care System, Palo Alto, CA, USA; 3 Stanford University Medical Center, Stanford, CA, USA; 4 Norwegian Center for Addiction Research, University of Oslo, Oslo, Norway

## Abstract

**Background:**

From a health services perspective, peer-based resources merit special attention. Participation in self-help fellowships, like the Twelve Step Groups (TSGs), have been shown to improve outcomes of patients with substance use disorder (SUD) and they represent a valuable adjunct to the SUD treatment system. This study investigated the relationship between patient perceptions of TSGs and the intent to participate in TSGs after receiving detoxification treatment.

**Methods:**

We included 139 patients that entered a detoxification unit (detox) in Kristiansand, Norway. We analyzed factors associated with the intention to participate in TSGs post-discharge with contingency tables and ordinal regression analysis.

**Results:**

Forty-eight percent of patients had participated in TSGs before entering detox. Respondents saw more advantages than disadvantages in TSG participation, but only 40% of patients showed high intentions of participating in TSGs post-discharge. A high intention to participate in TSGs was most strongly correlated with the notion that participation in TSGs could instill the courage to change. In a multivariate analysis, the perception that TSGs were beneficial was the strongest factor related to a high intention of TSG participation after treatment.

**Conclusions:**

Our findings increased the understanding of factors most likely to influence decisions to attend TSGs in SUD treatment contexts with uncommon TSG participation. Our results suggested that the majority of patients may be sufficiently influenced by highlighting the potential gains of TSG participation. Treatment programs that do not focus on self-help group attendance during and after treatment should consider implementing facilitative measures to enhance utilization of these fellowships.

## Background

Publicly funded health services are currently exposed to increasing fiscal constraints, and this may cause a reduction in service [1]. To remain abreast with ever increasing demands, one option for treatment services might be to consider greater involvement with voluntary services, like peer-based groups. This strategy is also recommended by the World Health Organization (WHO) [2,3]. Patients with substance use disorder (SUD) have the option to participate in addiction-focused, mutual-help groups, like the Twelve Step Groups (TSGs) of Alcoholics Anonymous (AA) or Narcotics Anonymous (NA). Participation in TSGs has been shown to improve success rates after treatment and TSGs are considered a valuable adjunct to formal SUD treatment [4-6]. These groups are valued as an important recovery resource in their country of origin, the United States. Accordingly, engagement strategies, most notably the Twelve Step Facilitation (TSF) initiative, have been developed for clinicians to introduce the 12-step principles to patients [7]. The prevalence of lifetime TSG attendance in the target population of patients with SUD is generally high in the U.S.; three studies reported that 66%, 78%, and 83% of patients had previously had at least some involvement at treatment arrival [8-10]. High levels have also been noted in the intention to participate in TSGs; in a recent U.S.-based study, 79% of patients planned to attend AA or NA at least twice a week after treatment [11]. Two United Kingdom studies reported similar pre-treatment attendance levels, with 73% and 77% of patients that had had at least some previous participation [12,13]. However, only 47% had intended to attend TSG meetings regularly following discharge [12].

In other European countries, studies on patient perceptions of TSGs are scarce. A few studies have investigated clinician attitudes and knowledge about TSGs; those findings suggested that clinicians were relatively unaware of these fellowships, and referrals were uncommon [14,15]. Hence, there is a need for developing strategies that enhance TSG utilization in areas that have little awareness of TSG programs. The first step is to examine substance users' experiences with and perceptions of existing 12-step fellowships.

### Factors Associated with TSG Participation

In early U.S.-based studies, the patient perceived severity of a SUD problem was the most reliable predictor of subsequent TSG participation [16,17]. Other demographic, personality, social, cognitive, or substance-related variables were weakly or inconsistently associated with participation [16]. The *Survey of Readiness for AA Participation *(SYRAAP) [18,19] indicated that there were three primary influential factors; the perceived severity of the SUD problem, the perceived benefits of TSGs, and the perceived barriers to TSG participation. These factors predicted TSG affiliation better than demographic or life context factors [19]. The SYRAAP may also be used to examine patient perceptions of TSGs. In the present analysis, we used behavioral intention as the dependent variable. This indexed a person's motivation to perform a particular behavior (here, to attend TSGs); it also indicated both the direction (i.e., yes/no) and intensity of a decision to engage in a behavior (e.g., the degree of effort a person is prepared to expend) [20].

### Objectives

The aims of this paper were: (1) Explore patient perceptions of the benefits and barriers of TSGs at admission to a detoxification unit (detox), and (2) investigate the relationship between patient perceptions of TSGs and the intention to participate in these groups following discharge.

## Methods

### Participants and procedures

Patients were recruited from a detox unit in the Addiction Unit, Sørlandet Hospital in Kristiansand, Norway from September 2008 to August 2010. The main service area for this institution was the most southern county in Norway (population 166, 000). Eligible patients had an alcohol or drug use disorder, did not receive opioid maintenance treatment, remained in the detox unit sufficiently long for assessment, were discharged to their homes, and had access to at least one TSG within 30 km of their home. Exclusion criteria were severe psychiatric co-morbidity and an inability to complete a structured interview (due to, for example, severe somatic symptoms, cognitive disability, or language problems). Of the 156 eligible patients, 16 refused to participate and one provided insufficient data. The final sample included 139 patients (89% of eligible respondents). After providing informed consent, participants completed the inventory described below. The Regional Ethics Committee of the South-East Health Region, Norway, approved the study.

### Measures

The Addiction Severity Index, European version (EuropASI), was used to collect data on patient demographics, life context, substance use, and treatment history [21,22]. The Mini International Neuropsychiatric Interview (MINI), version 5.0, was used to assess a SUD diagnosis [23]. Any pre-detox TSG affiliation was assessed with the AA Affiliation Scale *(AAAS) *[24]. The wording was modified to include both AA and NA. The frequency of attendance to 12-step meetings during one's lifetime and during the prior 6 months was coded with a 0 to 1 scale (e.g., the lifetime scale was: 0.25 = 1-30 meetings, 0.5 = 31-90 meetings, 0.75 = 91-500, and 1 = > 500 meetings). In addition, seven yes/no involvement items (e.g., read TSG literature, had a sponsor) were coded from 0 (no, never) to 1 (yes). The translated version of the AAAS had similar good internal consistency as the original scale (Cronbach's α = 0.81). The total TSG attendance and involvement scores resulted in a composite score that ranged from 0 to 9.

The *Survey of Readiness for AA Participation (SYRAAP) *measured the patients' perceived severity of the substance problem and the patients' perceptions of the relevance of TSGs to their problem. These were measured with the *perceived benefits *and *perceived barriers *items [19]. The wording was modified to include both AA and NA. Questions were rated in a 5-point Likert-type response format, where higher scores indicated higher levels of the construct under assessment. The Norwegian version had good internal consistency with Cronbach's α values between 0.75 and 0.85 for the three subscales [25]. To further confirm the psychometric properties of the scale after translation, a principal axis factor analysis was performed showing that the translated version had the same structure as the original version and all items had satisfactory factor loadings (> 0.4) within their respective sub-scales [26]. A mean score was computed for each subscale (5 questions in each subscale).

*The Intent to attend AA/NA *was rated with two questions on a 7-point Likert scale: "I intend to attend AA/NA meetings regularly (at least twice a month) over the next six months" and "I will attend AA/NA meetings regularly (at least twice a month) over the next six months" [27]. The two items were highly correlated (r = .98), and a composite score was computed by averaging them. For descriptive and analytic purposes, scale responses were categorized into low (< 3), moderate (3 - 5), and high (> 5) intentions.

The original English questionnaires (AAAS and SYRAAP) were translated to Norwegian with a standard procedure (two forward and two backward translations) [28], in collaboration with the questionnaire developers.

### Statistical analyses

For all variables, descriptive statistics were computed. The two translated scales were checked for reliability with Cronbach's α [25]. To examine the underlying factor structure of the SYRAAP, a principal axis factor analysis (with Promax rotation) was performed and compared with the original instrument [26]. In contingency tables, we used single items from the SYRAAP's benefits and barriers subscales to maximize information about these potentially modifiable constructs; the gamma measure of association was used to explore the strength of association between variables. Contingency table analysis and Chi-square or non-parametric tests (Kruskal-Wallis) were used to explore associations between the intention to participate in TSGs and independent variables. Finally, a multivariate ordinal regression analysis was performed; results were expressed as odd ratios (OR) and 95% confidence intervals (CI). Age, gender, and variables with p values < 0.2 in the bivariate analyses were included in the multivariate analysis [29]. The significance level was set at p < 0.05. All statistical analyses were performed with SPSS 16.0.

## Results

### Sample description

We studied 139 patients (Table 1), with a mean age of 41 years. One-third of the patients were women, almost all were native Norwegians or European-born, and about half were living alone. The majority of patients (77%) had had some kind of specialized SUD treatment previously, and nearly all had been diagnosed with alcohol and/or drug dependence (6 of the patients who were diagnosed with an alcohol use disorder only met the criteria for harmful use). Forty-eight percent of patients had previously participated in a TSG at least once. The mean AAAS composite score (TSG involvement) was 1.7 (out of a maximum of 9). The curve was highly positively skewed; 59% of respondents scored ≤1 (i.e., they did not respond "yes" to any involvement items).

**Table 1:** Characteristics of study respondents (N = 139)

Characteristic	N (%), or *Mean (SD)*
Age, years	*41 (14)*
Female	45 (32%)
Proportion native Norwegians or European origin	134 (96%)
Education, years	*11.2 (2.3)*
Relationship, proportion of singles	65 (47%)
Main diagnosis (ICD-10)	
Alcohol dependence (N = 48) or harmful use of alcohol (N = 6)	54 (39%)
Both alcohol and drug dependence	26 (19%)
Drug dependence	59 (42%)
Years of problematic use^a^, major drug/s of abuse	*11.4 (9.1)*
Earlier treatment (prior to current detox)	
No earlier treatment	32 (23%)
Outpatient treatment only	33 (24%)
Inpatient treatment	
12-step-based treatment	39 (28%)
Other inpatient treatment (detox or longer-term)	35 (25%)
Ever participated in TSGs before	66 (48%)
Earlier involvement in TSGs (AAAS composite score; scale 0 - 9)	1.7 (2.4)

^a ^Problematic use, as defined in EuropASI, was the consumption of 5 or more standard drinks at least 3 times weekly, or binge drinking on 2 coherent days to a level that afflicted daily functioning

### Perceived benefits and barriers associated with TSG participation

The two most frequently recognized benefits of participating in TSGs were "In AA/NA, I will find people who understand me" (78%), and "If I go to AA/NA, I will find people who can guide me in how to be sober" (73%) (Table 2). Between 55% and 78% of patients agreed with each of the five statements that described the potential benefits of TSGs; this suggested that the majority of patients thought that TSGs were a potential option for obtaining help and support with combating addiction. The two barrier statements most frequently recognized regarded embarrassment; first, they were embarrassed to go to AA/NA (37%), and second, they did not want other people to know that s/he was going to AA/NA (29%) (Table 2).

**Table 2:** Patient-perceived benefits and barriers towards TSGs in relation to their intention to participate in TSGs post-discharge, N (%)

*Items*	^a^	LOW^b^ N = 43	MOD^b^ N = 41	HIGH^b ^ N = 55	Total	Gamma^c^
*Perceived benefits*						
Going to AA/NA gives me courage to change (N = 138)	Disagree	11	0	0	11 (8)	
	N/N	24	19	6	49 (36)	0.79
	Agree	8	22	48	78 (57)	
If I go to AA/NA, I will find people who can guide me in how to be sober (N = 135)	Disagree	8	1	0	9 (7)	
	N/N	18	9	0	27 (20)	0.78
	Agree	16	31	52	99 (73)	
I will feel better about myself if I go to AA/NA (N = 139)	Disagree	16	1	2	19 (14)	
	N/N	21	17	6	44 (32)	0.71
	Agree	6	23	47	76 (55)	
In AA/NA, I will find people who understand me (N = 138)	Disagree	3	0	2	5 (4)	
	N/N	18	6	1	25 (18)	0.62
	Agree	21	35	52	108 (78)	
I know someone who has been helped by going to AA/NA (N = 138)	Disagree	19	10	5	34 (25)	
	N/N	6	9	3	18 (13)	0.51
	Agree	18	22	46	86 (62)	
*Perceived barriers*						
I feel like I do not belong at AA/NA meetings (N = 139)	Disagree	8	18	42	68 (49)	
	N/N	15	21	9	45 (32)	- 0.65
	Agree	20	2	4	26 (19)	
Going to AA/NA makes me feel depressed (N = 138)	Disagree	11	27	44	82 (59)	
	N/N	19	11	10	40 (29)	- 0.62
	Agree	12	3	1	16 (12)	
I do not want people to know that I am going to AA/NA (N = 139)	Disagree	10	22	37	69 (50)	
	N/N	10	11	9	30 (22)	- 0.43
	Agree	23	8	9	40 (29)	
Going to AA/NA requires changes that are too difficult (N = 139)	Disagree	9	14	33	56 (40)	
	N/N	19	20	13	52 (37)	- 0.41
	Agree	15	7	9	31 (22)	
Going to AA/NA can be embarrassing to me (N = 139)	Disagree	9	21	32	62 (45)	
	N/N	12	6	7	25 (18)	- 0.36
	Agree	22	14	16	52 (37)	

^a ^For descriptive purposes, the original scale has been coded pooling strongly agree and agree responses, and the strongly disagree and disagree responses. N/N = neither disagreed nor agreed

^b ^LOW, Low score < 3; MOD, intermediate score = 3 - 5; HIGH, high score > 5, on a 7-point Likert scale

^c ^Gamma-values were obtained from an analysis of the full ordinal scale for independent variables. All items were significant at the p < 0.001 level.

### Intention to participate in TSGs post-detox

The intention to participate regularly in TSGs during the 6 months following detox was low for 43 (31%) patients (score < 3), moderate for 41(29%) patients (score = 3-5), and high for 55 (40%) patients (score > 5).

### Patient perceptions associated with intention to participate in TSGs

There was a clear trend that patients that perceived more benefits and fewer barriers also had higher intentions to participate in TSGs post discharge (Table 2). However, those with high and moderate intentions also seemed to put some weight on embarrassment in going to AA/NA; about 3 in 10 agreed with that item. The strongest positive correlations were found in the constructs "Going to AA gives me courage to change" (gamma = 0.79, p < 0.001) and "If I go to AA/NA, I will find people who can guide me in how to be sober" (gamma = 0.78, p < 0.001). The strongest negative correlation was found for "I feel like I do not belong at AA/NA meetings" (gamma = - 0.65, p < 0.001; Table 2).

### Independent variables associated with Intention to participate in TSGs

Associations were analyzed between the mean values and proportions of independent variables and the intention to participate in TSGs after treatment (Table 3). The results indicated that women were more polarized than men in their intention to participate in TSGs. The TSG involvement scores (Table 3) and attendance rates (35%, 34%, and 67% in the 'low', 'moderate' and 'high' intention groups, respectively) indicated that patients with the highest prior attendance/involvement were the most inclined to re-enter the groups. The three SYRAAP subscales showed that patients with high perceived severity, high perceptions of benefits, and low perceptions of barriers had significantly higher intentions to participate in TSGs compared to the other patients. All three groups (high, moderate, and low intentions) had mean scores on the positive side of the TSG benefit subscale; i.e., more agreed than disagreed with the benefit items. The perceptions of benefits differed among groups only in strength. On the perceived barriers subscale, the mean scores for the low intention group was on the agreement side of the scale (i.e., more agreed than disagreed with barrier items); in contrast, both the moderate and high intention groups had mean scores on the disagreement side (i.e., more disagreed than agreed with barrier items). These findings suggested that there was a qualitative difference between the low versus the moderate and high intention groups in the perception of TSG barriers. Thus, the barriers were a problem primarily in the low intention group. No other factors, including age, relationship status, educational status, or diagnosis had significant influence on the intention to participate in TSGs. However, in contrast to patients with lower intentions, those with higher intentions tended to be older and to have an alcohol use disorder diagnosis (Table 3).

**Table 3:** Comparison between patient intentions to participate regularly in TSGs and independent variables

*Items*	LOW^a^ N = 43	MOD^a^ N = 41	HIGH^a^ N = 55	P-value^b^
Gender (female)	19 (44%)	7 (17%)	19 (35%)	0.027
Age, years	*40 (14)*	*38 (13)*	*44 (13)*	0.081
Relationship, % single	23 (54%)	19 (46%)	23 (42%)	0.516
Education, years	*11 (2)*	*11 (2)*	*12 (3)*	0.481
Main diagnosis				
Alcohol use disorder	13 (30%)	15 (37%)	25 (46%)	
Both alcohol and drug use disorder	7 (16%)	5 (12%)	14 (26%)	0.088
Drug use disorder	23 (54%)	21 (51%)	16 (29%)	
Perceived drug problem severity (SYRAAP subscale)	*3.9 (0.7)*	*4.2 (0.6)*	*4.3 (0.6)*	0.012
Perceived benefits of TSGs (SYRAAP subscale)	*3.1 (0.8)*	*3.7 (0.5)*	*4.3 (0.6)*	< 0.001
Perceived barriers of TSGs (SYRAAP subscale)	*3.3 (0.8)*	*2.6 (0.8)*	*2.2 (0.6)*	< 0.001
Earlier involvement in TSGs (AAAS composite score)	*0.8 (1.7)*	*0.9 (1.6)*	*3.0 (2.9)*	< 0.001

Data are the *mean (SD) *and N (%), N = 139.

^a ^LOW, Low score < 3; MOD, intermediate score = 3 - 5; HIGH, high score > 5, on a 7-point Likert scale

^b ^P-value was obtained with a Kruskal-Wallis or Chi-square test

Only two variables were retained in the multivariate analysis; the perceived barriers, which were negatively associated, and the perceived benefits, which were positively associated with high intentions to participate in TSGs after treatment (Table 4). The strongest influential factor was the perception that TSGs were beneficial (OR 4.74, 95% CI = 2.34 - 9.63, p < 0.001).

**Table 4:** Multivariate ordinal regression analysis of associations between the intention to participate in TSGs after detox treatment versus independent variables (N = 139)

*Independent variables*	OR	95% CI	P- value
Gender (women)	1.36	0.67 - 2.75	0.394
Age	0.98	0.95 - 1.02	0.274
Main diagnosis			
Drug dependence (reference)	-		
Both alcohol and drug dependence	1.30	0.50 - 3.40	0.595
Alcohol use disorder	2.68	0.97 - 7.41	0.058
Earlier involvement in TSGs (AAAS composite score)	1.13	0.96 - 1.34	0.142
Perceived drug problem severity	1.40	0.81 - 2.50	0.218
Perceived barriers towards TSGs	0.54	0.32 - 0.91	0.021
Perceived benefits of TSGs	4.74	2.34 - 9.63	< 0.001

Results are adjusted odd ratios (OR) and 95% confidence intervals (CI); age, gender and variables with p < 0.2 in bivariate analyses (Table 3) were included in the multivariate analysis.

## Discussion

This study found that less than half of the participating patients that entered the detox program in this Norwegian addiction treatment unit had previously attended TSGs. Nevertheless, three-quarters of patients agreed with the benefit items on the questionnaire. This implied an understanding that TSGs were a potential supportive resource. However, only 40% of patients reported a high intention to participate in TSGs after discharge. The constructs most strongly correlated with a high intention to participate in TSGs after detox were the notions that participation in TSGs could instill the courage to change and that TSGs could provide abstinence-specific support. The strongest predictor of a low intention to participate was the sense of not belonging in AA/NA. Between-group comparisons based on categorizations of low, moderate, and high intentions indicated that perceived barriers was a problem primarily in the low intention group. An ordinal regression model showed that the recognition of TSG benefits was the strongest influence on the intention to participate in TSGs after treatment.

Forty-eight percent of patients had participated in TSGs prior to admittance at the detox unit. This proportion was substantially lower than the 66% to 83% reported in previous U.S. and U.K.-based studies [8-10,12,13] and also lower than the 60% observed in a Swedish treatment study [30]. An even smaller proportion reported some involvement with these fellowships; only 41% had at least one positive response to the TSG involvement questionnaire items. The twelve-step based treatment models usually require patients to begin TSG attendance during treatment [31]. This study was conducted in a Norwegian county that offered a 12-step-oriented treatment unit; however, 12-step based treatment units are quite rare in Norway; they comprise less than 5% of the available treatment programs [32]. Therefore, lower TSG attendance rates could be expected in most other Norwegian counties. Thus, the present findings indicate that the TSG attendance rate in Norway is lower than that found in other European countries, including the U.K., and neighboring Sweden.

Despite the fact that a majority (52%) of patients had no prior TSG experience, a substantially larger proportion of patients agreed with the TSG benefit items than with the TSG barrier items. This suggested that perceptions of TSGs tended to be more positive than negative, and that TSGs were perceived as potential supportive resources. Although this Norwegian sample had less prior experience with TSGs than that observed in a U.S.-based patient sample (48% versus 66% had ever attended AA/NA) [9], a large proportion of the Norwegians (roughly three-quarters of respondents) recognized the benefits frequently cited by U.S. patients, including support and fellowship from peers and help with sobriety and recovery. An important mechanism in peer support groups is the sense of identification with other attendees. This feature may be appreciated even more than the support obtained from professionals [33]. Almost three-quarters of patients also agreed that TSGs were a possible resource for obtaining abstinence-specific support. Although being drug free is not required for attending TSGs, these fellowships are strongly abstinence-oriented [34]. Our patients seemed to perceive both that they needed support in achieving abstinence and that TSGs offered a structure that may facilitate the achievement of that goal.

The intention to participate in TSGs was most strongly correlated with the notion that participation in TSGs could instill the courage to change. Addiction researchers have underscored the chronic nature of SUDs [35]. Thus, patients have negative perceptions of coping with triggers and urges and are likely to experience relapses. Although the negative consequences of addiction may steer people towards making changes, hope and courage may be required before abstinence becomes a realistic alternative. Thus, identifying with role models that have learned to manage their addiction represents a positive adjunct to the support provided by professionals [36,37].

The most important negative predictor for intending to participate in TSGs was "I feel like I do not belong at TSG meetings". There are at least two reasons for agreeing with this item. The individual may have attended a TSG previously and decided that "this is not the right place for me". Another possibility is that they had not been to a TSG previously, or they had attended just a few meetings, but did not stay long enough to gain a sense of belonging to the group. To get involved with a new social group, one has to get past the difficult threshold of attending the first meeting, and persevere long enough to become accustomed to the new setting. Tonigan, Connors, and Miller (2003) highlighted the importance of initiating TSG attendance during formal treatment of individuals with addictions [38]. The Matching Alcoholism Treatments to Client Heterogeneity (MATCH) project compared patients that were/were not involved in TSGs during treatment. Those not involved during treatment had much less TSG participation after treatment. Thus, a 12-step facilitation initiative that encouraged patients to attend TSGs during treatment appeared to contribute significantly to patient participation rates post discharge [38].

One of the barriers recognized by those with high intentions to participate in TSGs was embarrassment about going to AA/NA. This may reflect the difficulty that many patients have in disclosing a problem with alcohol and drugs. For those that had attended previously, there may also be emotional and psychological obstacles related to rejoining a group after a relapse [31]. Thus, patients may require extra support and encouragement to attend a TSG when they are ambivalent about rejoining a group.

Forty percent of patients reported a high intention to participate in TSGs after discharge; 31% had a low intention; and 29% had a moderate intention. These findings were consistent with those of a U.K.-based study by Harris et al., who reported a similar distribution; roughly one-third of the sample fell into each category of "negative", "neutral", or "positive" attitudes toward TSGs [13]. Those authors noted that the presence of a considerably sized, non-polarized/neutral group challenged their preconception that substance users were heavily polarized in their attitudes towards TSGs [13]. These "neutral" individuals are likely to be moved towards a greater intention to participate when addiction professionals highlight the potential gains of participation and recommend participation. The present study supported these notions; our results indicated that the perceived barriers towards TSGs were a problem primarily in the low intention group. Recommendation from professionals may enhance patient perceptions of the relevance of TSGs to their problems; moreover, the clinician's approval of TSGs may be an important pathway towards recovery [39]. The importance of enhancing the perception of TSG benefits was also implied by our regression analysis, which demonstrated a strong association between a high perception of benefits and a high intention to participate in TSGs.

### Methodological considerations

This study was among the few European studies to examine patient perceptions of TSGs. The study had a number of strengths that included a focus on complementary peer-based resources for a population that had no or few continuing care appointments in the formal treatment system although they were likely to need support over lengthy intervals. Standardized instruments were used. The original English questionnaires (AAAS and SYRAAP) underwent a formal translation procedure to provide a level of quality for the validity of the questionnaire content [28]. The psychometric properties of the translated scales were satisfactory. Although slightly older (41 versus 38 years), the present sample was similar to the detox patients (N = 564) of a previous regional multisite study [40]; they exhibited similar gender, ethnicity, major substance of abuse, and previous SUD treatment. We have no reason to believe that the health region in this study differs from other regions in Norway. Thus, the findings of the present study were generalizable to other detox patients in Norway.

This study also had limitations, particularly in interpreting the findings. We used a cross-sectional design that did not allow the establishment of cause among the variables. In addition, the dependent variable in the analysis was a psychological construct (behavioral intention) known to predict behavior, but we had not established to what degree patients actually follow their intentions. However, an intention is regarded as the most immediate and important predictor of subsequent behavior [20]. Although we measured perceptions of TSGs with a standardized instrument, it was developed in a different cultural setting and the list of single items most relevant for the perceived benefits/barriers constructs may not be exhaustive in the present culture.

### Implications

The findings of this study have increased the understanding of the beliefs likely to influence the patient's decision to attend TSGs after SUD treatment in contexts where TSG attendance and involvement are not routine. These modifiable perceptions may be targeted by clinicians to promote patient readiness to participate in TSGs. Treatment programs not accustomed to putting a focus on self-help group attendance during and after treatment should consider implementing facilitative measures. For example, members of AA/NA could be invited to treatment units to acquaint patients with the groups.

## Conclusions

Overall, our findings suggested that a majority of patients could potentially be motivated to attend TSGs with a relatively simple strategy. The primary strategy should be to highlight the potential gains of participation. Those hesitant in joining the TSGs should be encouraged to explore potential barriers that give rise to scepticism towards TSGs. Acquainting patients with TSGs may reduce perceived barriers and enhance utilization of these voluntary fellowships. Patients with no or little continuing care in the formal services should be made aware of these informal, accessible recovery resources.

## Competing interests

The authors declare that they have no competing interests.

## Authors' contributions

JKV participated in designing the study, collecting data, and interpreting results; in addition, JKV performed the analysis, and drafted the manuscript. CT, ØK, and TC participated in designing the study, interpreting results, and drafting the manuscript. All authors read and approved the final manuscript.

## Pre-publication history

The pre-publication history for this paper can be accessed here:

http://www.biomedcentral.com/1472-6963/11/339/prepub
